# Curcumin Supplementation Improves Gastrointestinal Symptoms in Women with Severe Obesity: A Double-Blind, Randomized, Placebo-Controlled Trial—A Pilot Study

**DOI:** 10.3390/nu17132064

**Published:** 2025-06-20

**Authors:** Fabiana Martins Kattah, Nayra Figueiredo, Kamilla Kenned Bezerra, Emilly Santos Oliveira, Cinara Costa de Melo, Gislene Batista Lima, Jocélia Paula Rocha Cavalcante, Beatriz Bacheschi do Carmo Benetti, Glaucia Carielo Lima, João Felipe Mota, Flávia Campos Corgosinho

**Affiliations:** 1 Faculty of Nutrition, Federal University of Goiás, Goiânia 74605-080, Brazil; fabianakattah@discente.ufg.br (F.M.K.); kamillakenned@discente.ufg.br (K.K.B.); emillysantos@discente.ufg.br (E.S.O.); gislenelima@discente.ufg.br (G.B.L.); nutri.joceliarocha@gmail.com (J.P.R.C.); glauciacarielo@ufg.br (G.C.L.); 2 Faculty of Medicine, Federal University of Goiás, Goiânia 74605-080, Brazil; nayrafgrdo@hotmail.com (N.F.); cinara.melo@discente.ufg.br (C.C.d.M.); beatrizbenetti@discente.ufg.br (B.B.d.C.B.)

**Keywords:** gastrointestinal symptoms, stool consistency, turmeric

## Abstract

Gastrointestinal symptoms, including reflux, bloating, dyspepsia, stomach pain, and altered bowel patterns, are commonly reported in individuals with severe obesity and may significantly impact quality of life. **Background/Objectives**: Curcumin, a bioactive compound found in turmeric (*Curcuma longa* L.), possesses anti-inflammatory and antioxidant properties and has been investigated for its potential role in gastrointestinal health. However, its effects in individuals with severe obesity remain unclear. **Methods**: This double-blind, placebo-controlled clinical trial aimed to evaluate the effect of curcumin supplementation on gastrointestinal symptoms in women with severe obesity. Thirty-one women with a body mass index (BMI) ≥ 40 kg/m^2^ undergoing bariatric surgery were randomized to receive either 1500 mg of curcumin (98.75%) or a placebo (1500 mg corn starch) daily for 13 weeks. Gastrointestinal symptoms were assessed using the Gastrointestinal Symptom Rating Scale (GSRS), and stool consistency was assessed using the Bristol Stool Scale. Anthropometric measurements were also collected. **Results**: Participants had an average age of 33.1 ± 8 years and a BMI of 45.6 ± 3.31 kg/m^2^. No differences were observed between groups at baseline. At the end of the study, the curcumin group showed a significant reduction in the GSRS’s total score compared to the placebo group (*p* = 0.002), with improvements in eructation (*p* = 0.011) and constipation (*p* = 0.007). Additionally, the curcumin group showed reduced BMI (*p* = 0.019) and neck circumference (*p* = 0.042). **Conclusions**: These findings suggest that curcumin supplementation may alleviate some gastrointestinal symptoms and improve anthropometric measures in women with severe obesity, providing a potential dietary strategy.

## 1. Introduction

Obesity is a complex, multifactorial disease characterized by excessive adiposity, which increases the risk of many comorbidities [1]. Among its many health complications, obesity is strongly associated with gastrointestinal symptoms and complications, including defecatory dysfunction [2]. The prevalence of fecal incontinence is significantly higher in patients with obesity compared to those with normal weight, reaching up to 68% [3]. Constipation affects between 17.2% and 29.4% of this population, while diarrhea can occur in up to 40% of cases [3]. Additionally, alterations in gastrointestinal function can lead to nutritional deficiencies in this population [2]. Individuals with obesity also exhibit increased intestinal permeability to bacterial products such as lipopolysaccharide, which contribute to systemic inflammation [4].

In cases of severe obesity (body mass index ≥ 40 kg/m^2^), more invasive interventions such as bariatric surgery are often indicated [5]. However, some studies suggest that gastrointestinal symptoms may worsen following surgery, likely due to anatomical and functional changes in the digestive system [6,7]. Furthermore, patients who present with gastrointestinal symptoms before surgery may be at risk of persistent or exacerbated symptoms postoperatively [8].

Given these challenges, alternative therapeutic strategies have gained attention, particularly bioactive compounds with the potential to improve gastrointestinal health without the adverse effects associated with pharmacological treatments [9]. These compounds can act as prebiotics, increasing the production of short-chain fatty acids (SCFA) and modulating the composition of intestinal microbiota [10].

Curcumin, the main curcuminoid present in turmeric (*Curcuma longa* L.), has demonstrated multiple biological activities, including anti-inflammatory, anticancer, and antioxidant properties [11]. In vitro, curcumin promoted the growth of beneficial bacteria such as *Lactobacillus rhamnosus* GG (LGG) and *Bifidobacterium animalis* (BB12), suggesting a potential prebiotic effect [12]. In animal models, curcumin has also been associated with increased microbial diversity in the gut [13]. Consistent with these findings, polyphenols have been postulated as being duplibiotic and as agents capable of promoting beneficial microbial species through both antimicrobial and prebiotic effects [14]. One of the key mechanisms underlying these benefits may be the modulation of intestinal permeability and the reduction in metabolic endotoxemia, thereby attenuating inflammatory signaling [15].

A pilot study demonstrated that eight weeks of curcumin supplementation improved symptoms of irritable bowel syndrome in healthy adults [16]. Despite the high prevalence of gastrointestinal disturbances among individuals with obesity, evidence regarding the impact of bioactive compounds, particularly curcumin, on gastrointestinal function in this population remains limited. Therefore, this study aimed to evaluate the effect of curcumin supplementation on gastrointestinal symptoms in women with severe obesity prior to bariatric surgery.

## 2. Materials and Methods

### 2.1. Subjects

Women with severe obesity (BMI ≥ 40 kg/m^2^), aged between 20 and 59 years, from the Obesity Control and Surgery Program (PCCO) at the Hospital Estadual de Goiânia Doutor Alberto Rassi were included in this study. Women with autoimmune diseases, acute inflammatory diseases, genetic syndrome, inflammatory bowel disease, cancer, or chronic alcohol consumption were excluded.

This study was conducted in accordance with the Declaration of Helsinki and approved by the Research Ethics Committee of the Federal University of Goiás (n. 3.251.178) and the Hospital Estadual de Goiânia Doutor Alberto Rassi (n. 961/19). This study was also registered on Rebec (RBR-22pqs9). Written informed consent was obtained from all study participants before study enrollment.

### 2.2. Experimental Design and Randomization

This study is a randomized, double-blind, placebo-controlled trial. Women were invited to participate during their initial consultation with the surgeon. Upon providing consent, they were randomly assigned by an external researcher to either the placebo group (PG) or the curcuminoid group (CG). The PG received cornstarch capsules, while the CG received capsules containing curcumin (97.85%).

At their first appointment with the hospital’s registered dietitian, participants underwent assessments of anthropometric parameters, dietary intake, and gastrointestinal symptoms. Following these evaluations, they were provided with the assigned supplement for 13 weeks. Since curcumin presents low bioavailability and is fat-soluble [17], patients were instructed to take three capsules after lunch and dinner, meals with a higher fat content. Each capsule contained 250 mg of curcumin, totaling six capsules per day (1500 mg/day).

**Figure 1:**
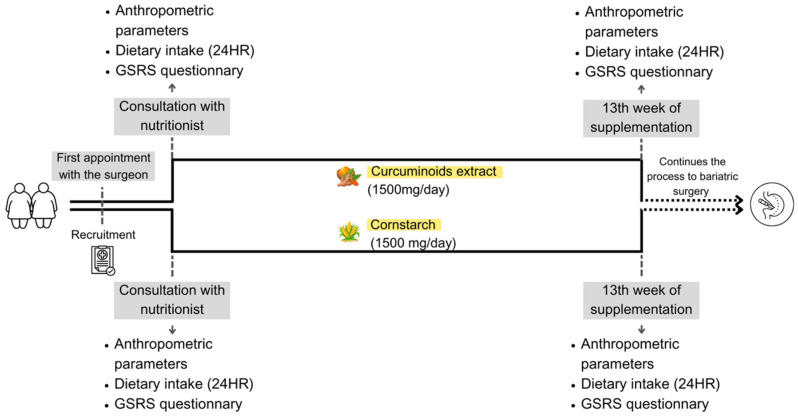
Study design.

At the end of the intervention period, the same assessments were repeated. The study design can be seen in Figure 1. Throughout the study, all participants, from both groups, were encouraged to adhere to the guidelines provided by the multidisciplinary PCCO team, which included the surgeon, nutritionist, psychologist, and endocrinologist.

### 2.3. Total Score of Gastrointestinal Symptom Rating Scale (GSRS) 

The GSRS was used, which is the most appropriated tool since it requires a short time to be applied, it is easy to use, and it refers to a recent period (previous week) [18]. The GSRS is a questionnaire with 15 items that measure the severity of gastrointestinal symptoms. Questions evaluate abdominal pain, reflux, diarrhea, indigestion, and constipation. Responses are rated on a 7-point scale, which varies from no discomfort (1) to very severe discomfort (7) [18]. The questionnaire was applied before and after supplementation and could evaluate gastrointestinal symptoms, as performed in previous studies [19,20]

### 2.4. Anthropometric and Food Consumption Assessment

The anthropometric assessment consisted of measuring weight and waist (WC), hip (HC), and neck (NC) circumferences. To measure weight, the women stood in light clothing and barefoot on a scale (P-200M Líder, Araçatuba, Brazil) with a capacity of 200 kg. Waist circumference was taken at the height of the navel when possible or at the largest protuberance observed (Pereira et al., 2023 [21]). Hip circumference was obtained from the widest part of the hips, and neck circumference was measured below the laryngeal prominence [22]. The waist-to-hip ratio was calculated according to validated measurements [23].

Food consumption was assessed using the 5-step multiple pass method in a 24 h recall [24]. Food records were calculated using Avanutri, online version. Calorie intake and macronutrient intake (in percentages) were evaluated. Although this study did not carry out an intervention on food consumption, it is important to note that those women were undergoing bariatric surgery and were accompanied with the hospital’s nutritionist.

### 2.5. Statistical Analysis

Statistical analyses were conducted using Jamovi version 2.3 (The Jamovi Project, 2022). A post hoc power analysis was performed using G*Power v3.1.9.7 based on a two-tailed independent samples *t*-test (based on Delta values). Considering an observed effect size of *d* = 1.42, α = 0.05, and *n* = 12 per group, the achieved statistical power was 1 − β = 0.64 (noncentrality parameter δ = 2.449; degrees of freedom = 22; critical *t* = ±2.0739). Descriptive statistics are presented as mean ± standard deviation for normally distributed continuous variables, and as median (minimum–maximum) for non-normally distributed data. Normality was assessed using the Shapiro–Wilk test. Delta values (Δ) were calculated to represent intra-individual changes from baseline to follow-up. Between-group comparisons of delta values were analyzed using independent *t*-tests or Mann–Whitney U tests depending on normality. Within-group changes over time were assessed using paired *t*-tests or Wilcoxon signed-rank tests. For intergroup comparisons post-intervention, an analysis of covariance was used to adjust for potential confounders: BMI, protein intake, and carbohydrate intake. After these adjustments, the results remained statistically significant, indicating that these variables did not influence the outcome. Additionally, Cohen’s *d* effect size was calculated to assess the clinical relevance of the observed changes, interpreted according to conventional thresholds: small (*d* = 0.2), medium (*d* = 0.5), and large (*d* ≥ 0.8). The level of statistical significance was set at *p* < 0.05.

## 3. Results

### Sample Description

**Figure 2:**
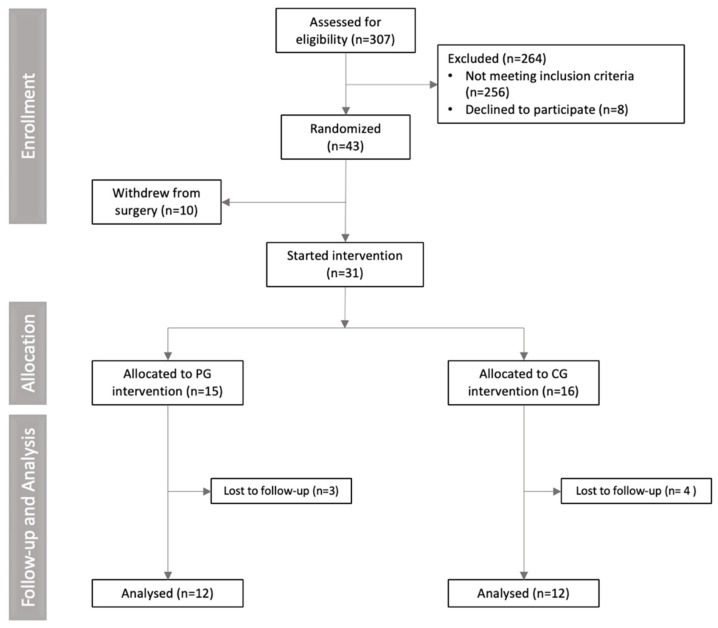
CONSORT flowchart diagram of clinical trial.

A total of 307 volunteers were assessed for eligibility. Of these, 274 were excluded for not meeting the inclusion criteria (n = 256), declining to participate (n = 8), or withdrawing from surgery (n = 10). Consequently, 31 participants were randomized into the curcumin or placebo groups and completed the study. A total of seven participants withdrew from the study. The reasons for dropout in the placebo group (n = 3) were no longer wanting to take capsules (n = 1), hemorrhage related to menstrual cycle (n = 1), and failure to attend the second meeting (n = 1). In the supplemented group, the reasons for withdrawing from the study (n = 4) were personal reasons (n = 1), discovered cholelithiasis (n = 1), and difficulty taking capsules (n = 2). No participants left the study because of side effects. The CONSORT flowchart diagram is presented in Figure 2.

The baseline characteristics of the participants are presented in Table 1. The average age of the sample was 33 ± 8 years, and the anthropometric values were similar between groups at baseline. As expected in a population with severe obesity, women presented higher neck and waist circumference values. Gastrointestinal symptoms, as assessed by the GSRS, and stool consistency, based on the Bristol Stool Chart, did not differ between groups at the beginning of the study.

After 13 weeks of supplementation, participants in the curcumin group showed a significant reduction in gastrointestinal symptoms. The total GSRS score decreased by 7.36 points (*p* = 0.003; *d*= −1.17), and these improvements remained significant after adjusting for BMI (*p* = 0.014; *d* = 0.017), carbohydrate intake (*p* = 0.002; *d* = 1.130), and protein intake (*p* = 0.001). Specific symptoms that improved included eructation (*p* = 0.011; *d* = −0.858) and constipation (*p* = 0.007; *d* = 0.562) (Table 2). No significant changes in gastrointestinal symptoms were observed in the placebo group. Additionally, stool consistency did not significantly change in either group.

Regarding anthropometric outcomes, a significant reduction in BMI (−4.0 kg/m^2^; *p* = 0.019; *d* = 0.017) and neck circumference (*p* = 0.042; *d* = 0.440) were observed in the curcumin group. These changes were not observed in the placebo group (Table 1).

Although the placebo group showed an increase in protein intake (*p* = 0.002) and a reduction in carbohydrate intake (*p* = 0.002) after the intervention, no significant differences in dietary intake were detected between groups at baseline or post-intervention (Table 3).

## 4. Discussion

The present study demonstrated, for the first time, that curcumin supplementation alleviates gastrointestinal symptoms in women with severe obesity. Even though it is an exploratory and preliminary study, it is necessary to consider that gastrointestinal symptom complaints are common in individuals with class III obesity [8] and are known to negatively impact quality of life [25]; thus, identifying strategies that improve gastrointestinal health in this population is of particular relevance.

In our study, curcumin supplementation reduced self-reported eructation. This finding aligns with prior research indicating the potential benefits of curcumin in functional dyspepsia [25]. Moreover, a narrative review reported that curcumin, when used as an adjuvant therapy for as *Helicobacter pylori* infection, a condition often associated with bloating and eructation, enhanced eradication rates [26].

We also observed a moderate reduction in self-reported constipation, although no changes were detected in stool consistency as assessed using the Bristol Stool Scale. This partially contrasts with a previous study conducted in Australian adults with digestive disorders in which a herbal supplement containing a low dose of curcumin (30.37 mg) improved stool consistency towards the ideal type 4 [27]. While preclinical models suggest that curcumin may modulate gut motility and relieve both constipation and diarrhea [28], further research is needed to clarify its effects on intestinal transit and motility in humans. It is important to note that curcumin supplementation did not result in significant improvements in the other gastrointestinal parameters evaluated, including abdominal pain, heartburn, acid regurgitation, hunger pains, nausea/vomiting, borborygmus, bloating, flatulence, hard stools, diarrhea, the urgency to defecate, and a sensation of incomplete evacuation.

The selective improvement in eructation and constipation but not in other symptoms may be attributed to curcumin’s potential effect on motility and microbial composition, which more directly influence gas production and intestinal transit [13,28]. In contrast, other symptoms involve more complex or distinct pathophysiological mechanisms, such as visceral hypersensitivity, altered gastric acid secretion, and neuromodulation, which may not be sufficiently affected by the dose or duration used in this study, as suggested by preclinical findings [29,30].

Evidence from a pilot study shows that turmeric standardized extract (144 mg/day) reduced the prevalence of irritable bowel syndrome symptoms over an eight-week period [16]. Additionally, curcumin, when combined with conventional pharmacological therapy for inflammatory bowel diseases, has been associated with improved stool frequency, consistency, abdominal pain, and remission rates [31]. These benefits may be related to the anti-inflammatory properties of curcumin, which include the suppression of key mediators such as tumor necrosis factor α (TNF-α), nuclear factor kappa B (NF-KB), and interleukin-1 B (IL-1B), potentially through the p38 mitogen-activated protein kinase (MAPK)-dependent pathway [28,32].

Furthermore, emerging evidence suggests that curcumin may exert prebiotic-like effects. In vitro studies have shown stimulation of the growth of beneficial bacteria, such as *Lactobacillus rhamnosus* GG (LGG) and *Bifidobacterium animalis* (BB12) [12]. In animal models, curcumin has been linked to higher diversity and an increased production of short-chain fatty acids (SCFA), a group of microbial metabolites known to promote intestinal health and regulate gut motility [13]. Although we did not measure microbiota composition or SCFAs in this study, these mechanisms may contribute to the observed improvements in GI symptoms and warrant investigation in future trials.

Beyond gastrointestinal benefits, we also observed a small reduction in BMI in the curcumin-supplemented group. Although previous systematic reviews have reported reductions in BMI with curcumin supplementation in patients with obesity, polycystic ovarian syndrome, non-alcoholic fatty liver disease, and metabolic syndrome [33,34], to our knowledge, this is the first study to report such an effect in a population with class III obesity. The discrepancy between our findings and those from studies that failed to show BMI changes may be due to differences in study duration (~12 weeks), population characteristics, and curcumin dose (~1000 mg) [35,36,37,38,39,40].

Experimental evidence suggests that curcumin can attenuate adipocyte hypertrophy and reduce adipose tissue accumulation [13], effects that appear to be mediated by gut microbiota-derived metabolites such as SCFA and succinate, which in turn lead to the activation of thermogenic genes [13]. Therefore, the observed BMI reduction may reflect, at least in part, microbiota-mediated mechanisms. Interestingly, we also observed a decrease in NC, an important marker of cardiometabolic risk [41], although no change was observed in waist circumference. To date, no studies have specifically assessed NC in response to curcumin supplementation, showing that this is a novel finding.

Dietary intake patterns changed in the control group compared to baseline but became more similar to the curcumin group by the end of the study. Despite these changes, overall caloric restriction was similar between groups, as recommended in preoperative care [21]. Fiber intake did not differ between groups and remained below the recommended levels for women (25–32 g/day) [42], which is consistent with previous findings in individuals with overweight, obesity, and type 1 diabetes [43]. This convergence in dietary intake supports the notion that the observed benefits in the curcumin group were not merely due to differences in diet composition but likely reflect the specific effects of the supplement.

The strengths of our study include originality in targeting a population with severe obesity and the use of a higher dose and longer duration of curcumin supplementation than many prior studies. However, some limitations should be acknowledged. First, curcumin’s poor bioavailability may have limited its effects; we attempted to mitigate this by instructing participants to consume the supplement after meals presenting a higher amount of fat (i.e., lunch and dinner). Second, we did not investigate gut microbiota composition, intestinal biomarkers, or microbial metabolites, which would have strengthened our mechanistic interpretations. Third, participants were engaged in multidisciplinary preoperative care, including dietary counseling and lifestyle intervention, which can alter gastrointestinal symptoms. Although it is not possible to assert that those results are exclusively due to curcumin supplementation, the statistical analysis compared the group receiving curcumin with the placebo group, showing that curcumin helped to mitigate symptoms. Also, the small sample size is a limitation, and the conclusions should be interpreted with caution. Given this limitation, we considered this research as a pilot study. Finally, this study is a sub-study of a broader clinical trial focused on adipose tissue outcomes; therefore, participants were not recruited based on the presence of gastrointestinal symptoms, although these are common in this population.

## 5. Conclusions

Curcumin supplementation improves some gastrointestinal symptoms, specifically reducing eructation and moderately alleviating constipation. In addition, supplementation led to a small reduction in BMI and a moderate reduction in NC. These findings suggest that curcumin supplementation may be a promising adjunctive strategy in the management of severe obesity.

## Figures and Tables

**Table 1:** Characterization of women with severe obesity at baseline.

	Placebo Group				Curcumin Group						
	Baseline (n = 12)	Follow-Up (n = 12)	Delta	*p* Value ^†^	Baseline (n = 12)	Follow-Up (n = 12)	Delta	*p* Value ^†^	*p* Follow-Up ^§^	*p* Value Delta ^§^	Cohen’s *d* /Biennial ^‡^
Age	36.09 ± 8.38	36.09 ± 8.38	-	1.000	31.5± 6.38	31.5± 6.38	-	1.000	0.205	-	-
BMI (kg/m^2^)	45.03 (41.6–50.9)	45.29 (40.72–50.9)	0.268	0.832	48.35 (42.3–52.9)	44.1 (41.6–50.3)	−1.67	0.273	0.895	**0.019**	**0.017**
Weight (kg)	117.4 (103–127)	115 (105.6–124	−12.45	0.660	119.9 (90.3–143)	116.7 ± 91.7–144.1	−3.83	0.645	0.608	0.207	0.236
Height (m)	1.59 (1.52–1.65)	1.59 (1.52–1.65)	-	1.000	1.61 ± (1.47–1.74)	1.61 ± (1.47–1.74)	-	1.000	-	0.205	-
WC (cm)	123.6 ± 10.7	122.3 ± 11.02	−12.72	0.787	129.6 ± 8.57	126.04 ± 10.52	−3.58	0.371	0.422	0.322	−0.228
NC (cm)	37 (34.7–48)	39 (36–45.5)	13.86	0.147	39.8 (36–46.8)	40.2 (33–45.5)	−0.06	0.954	0.307	**0.042**	**0.440**
HC (cm)	135.9 ± 10.5	138 ± 9.3	29.09	0.639	138.7 ± 7.43	137.7 ± 8.12	−0.11	0.665	0.769	0.498	0.194
GSRS	28.3 ± 6.40	30.25 ± 8.09	1.917	0.323	33.82 ± 9.90	30.25 ± 8.09	−7.36	**0.004**	0.253	**0.003**	**−1.17**
Bristol Stool Chart	4 (2–7)	5 (3–7)	0.1	0.881	4 (2–7)	4 (3–6)	−0.1	0.866	0.324	0.842	0.010

BMI: Body Mass Index; WC: Waist Circumference; NC: Neck Circumference; HC: Hip Circumference; GSRS: Gastrointestinal Symptom Rating Scale. *p* ≤ 0.05 was considered statistically significant; ^†^ T paired test; ^§^ T test or Mann–Whitney test; ^‡^ analysis regarding *p* value of delta. Bold represents statistically significant variables.

**Table 2:** Gastrointestinal Symptom Rating Scale (GSRS) score in control and curcumin groups.

	Placebo			Curcumin					
	Baseline (n = 12)	Follow-Up (n = 12)	Delta	Baseline (n = 12)	Follow-Up (n = 12)	Delta	*p* Value Follow-Up ^§^	*p* Value Delta ^§^	Cohen’s *d*/ Biennial ^‡^
Abdominal pain	1 (1–6)	1 (1–4)	0.45	1 (1–7)	1 (1–6)	−0.91	0.888	0.126	0.284
Heartburn	1 (1–3)	1 (1–5)	0.00	2 (1–7)	1 (1–7)	−10.0	0.701	0.358	0.104
Acid regurgitation	1 (1–2)	1 (1–7)	0.36	1 (1–7)	1 (1–7)	−0.66	0.402	0.240	0.250
Hunger pains	1 (1–4)	1 (1–5)	0.00	1 (1–5)	1 (1–4)	−0.08	0.716	0.848	0.000
Nausea/vomiting	1 (1–3)	1 (1–7)	0.36	1 (1–6)	1 (1–7)	−10.00	0.674	0.365	0.194
Borborygmus	2 (1–7)	1 (1–5)	−0.54	3 (1–7)	1 (1–3)	−18.33	0.325	0.320	−0.434
Bloating	1 (1–4)	1 (1–4)	−0.27	1 (1–1)	1 (1–4)	0.66	0.375	0.102	0.828
Eructation	3 (1–7)	4 (1–7)	0.90	3 (1–6)	2 (1–4)	−10.00	0.016	**0.011**	**−0.858**
Flatulence	4 (1–7)	4 (1–7)	0.09	4.5(1–7)	2.5 (1–7)	−14.16	0.330	0.162	−0.436
Hard stools	1 (1–2)	1 (1–1)	−0.09	1 (1–7)	1 (1–4)	−0.75	0.186	0.284	0.187
Constipation	1 (1–5)	1 (1–7)	10.91	1 (1–7)	1 (1–1)	−12.50	0.066	**0.007**	**0.562**
Loose stools	1 (1–7)	1 (1–7)	0.72	1 (1–7)	1 (1–4)	−0.66	0.082	0.278	0.291
Diarrhea	1 (1–5)	1 (1–2)	−0.36	1 (1–4)	1 (1–4)	−0.16	0.045	0.968	−0.006
Urgent need to defecate	1 (1–4)	1 (1–2)	−0.63	1 (1–6)	1 (1–4)	−0.41	0.608	0.765	−0.062
Incomplete evacuations	1 (1–6)	1 (1–6)	0.81	1 (1–5)	1 (1–5)	−0.50	0.162	0.107	0.333

Data are presented as median (minimum–maximum). *p* ≤ 0.05 was considered statistically significant. ^§^ T test or Mann–Whitney test; ^‡^ analysis regarding *p* value of delta. Bold represents statistically significant variables.

**Table 3:** Food consumption in placebo and curcuminoid groups.

	Placebo				Curcumin						
	Baseline (n = 12)	Follow-Up (n = 12)	Delta	*p* Value ^†^	Baseline (n = 12)	Follow-Up (n = 12)	Delta	*p* Value ^†^	*p* Value Follow-up ^§^	*p* Delta ^§^	Cohen’s *d*/Biennial ^‡^
Energy (kcal)	1345 ± 744	1175 ± 406	−170.2	0.516	1655 ± 1310	1130 ± 360	−234.8	0.190	0.672	0.837	−0.077
Carbohydrate (%)	52.9 ± 9.77	39.67 ± 8.07	−13.2	**0.002**	48.1 ± 10.1	47.4 ± 10.6	0.91	0.873	0.131	**0.008**	**1.130**
Protein (%)	15.58 ± 11.1	25.1 ± 10.3	9.25	**0.002**	21.1 ± 11.1	22.2 ± 6.83	−0.41	0.701	0.482	**0.001**	**−1.163**
Lipid (%)	31.5 ± 8.47	33.2 ± 9.25	1.69	0.666	30.8 ± 8.66	30.6 ± 6.57	−0.24	0.953	0.538	0.708	0.003
Total fiber	8.72 (3.0–30.9)	9.20 (3.4–13.3)	−1.69	0.847	10.6 (3.8–34.1)	8.64 (3.9–12.9)	−1.62	0.364	0.952	0.903	−0.060

Values are expressed as mean ± standard deviation of mean or median (minimum–maximum). *p* ≤ 0.05 was considered statistically significant. ^†^ Paired T test; ^§^ T test or Mann–Whitneytest; ^‡^ analysis regarding *p* value of delta. Bold represents statistically significant variables.

## Data Availability

The data presented in this study are available upon request from the corresponding author due to ethical reasons.

## Abbreviations

The following abbreviations are used in this manuscript:

BMI	Body mass index
CG	Curcumin group
GSRS	Gastrointestinal Symptom Rating Scale
PG	Placebo group
WC	Waist circumference
HC	Hip circumference
NC	Neck circumference
